# Clinical analysis of a new multifunctional instrument set for gasless endoscopic thyroidectomy with two different approaches

**DOI:** 10.1007/s00464-024-10678-1

**Published:** 2024-02-12

**Authors:** Bo Hu, Yuqing Chen, Yannan Jin, Xianfu Liu, Yansong Chen, Jingwei Tang, Yuan Liu, Zhe Zhang, Nanhai Wang, Ru Bai, Gongsheng Jin

**Affiliations:** 1 Department of Oncological Surgery, The First Affiliated Hospital, Bengbu Medical College, Anhui, China; 2 Queen Mary School, Nanchang University, Nanchang, China; 3 Department of Anesthesiology, The First Affiliated Hospital, Bengbu Medical College, Anhui, China; 4 Department of Oncological Nursing Surgery, The First Affiliated Hospital, Bengbu Medical College, Anhui, China

**Keywords:** Multifunctional instruments, Gasless endoscopic thyroidectomy, Thyroid carcinoma

## Abstract

**Background:**

Following the rapid development of endoscopic thyroidectomy techniques, various surgical procedures have been developed (e.g., transoral, submandibular, areolar, axillary, retroauricular, and combined procedures), and each of these procedures has its own advantages. In recent years, gasless endoscopic thyroidectomy has emerged as a feasible procedure, and it has replaced traditional CO2 insufflation approaches because of advantages such as stable cavity construction, pollution reduction, resource saving, and risk reduction. However, each gasless procedure requires special instruments for cavity construction, and this results in enormous wastage of medical resources. In the present study, we introduced a set of instruments developed by our team. This set of instruments is designed to be compatible with the current gasless endoscopic thyroidectomy approaches, including transoral, submandibular, transareolar, transaxillary, retroauricular, combined, and lateral cervical lymph node dissection. Here, we introduced this set of instruments for two gasless endoscopic thyroidectomy procedures (transaxillary and transareolar). Following the incorporation of this set of instruments in regular clinical practice, it could be used for more gasless endoscopic thyroidectomy procedures in the future.

**Objective:**

To investigate the feasibility, safety, and efficacy of the self-developed instruments for gasless endoscopic thyroidectomy in two different approaches.

**Methods:**

A total of 180 patients diagnosed to have papillary thyroid carcinoma (PTC) between January 2020 and April 2022 were retrospectively investigated. The patients were assigned to a gasless transaxillary group (group A) and a gasless transareolar group (group B). The same gasless endoscopic-assisted instruments were used for both groups. The clinical characteristics, treatment results, and complications were compared between the two groups.

**Results:**

All 180 patients were successfully operated. The extent of surgical resection in all patients was the same: “unilateral glandular lobectomy + isthmus combined with ipsilateral central zone lymph node dissection.” There were 130 and 50 patients in group A and group B, respectively; one patient in the former group was converted to open surgery due to intraoperative bleeding. No significant difference was observed between the two groups in terms of gender, age, body mass index (BMI), education level, and proportion of concomitant Hashimoto’s thyroiditis (P > 0.05). The establishment of cavity time was significantly longer in group A than in group B (35.62 ± 5.07 min vs. 17.46 ± 2.55 min, P < 0.01). The number of lymph nodes cleared was slightly less in group A than in group B (4.06 ± 2.93 vs. 4.52 ± 2.38, P = 0.07). Moreover, the two groups showed no significant differences (P > 0.05) in the total operative time (145.54 ± 45.11 min vs. 143.06 ± 46.70 min), tumor size (0.68 ± 0.46 cm vs. 0.71 ± 0.49 cm), postoperative hospital stay (4.08 ± 1.48 days vs. 3.72 ± 1.07 days), vocal cord paralysis [4 (3.1%) vs. 2 (4%)], postoperative swallowing discomfort [24 (18.5%) vs. 5 (10%)], and postoperative recurrence and satisfaction scores (3.27 ± 1.52 vs. 3.28 ± 1.53).

**Conclusion:**

Although the two approaches of gasless endoscopic surgery have different operative paths and different time periods for cavity construction, both approaches are similar in terms of the principle of cavity construction, safe and reliable postoperative efficacy, and good cosmetic effect. Therefore, the same set of instruments can be used to complete the surgery in both approaches, thus saving medical resources and facilitating the popularization of this technology.

The incidence of thyroid tumors has been increasing rapidly over the last decade, and these tumors are now recognized as one of the fastest-growing solid tumors. To date, surgery is the predominant method of treating thyroid tumors. The neck scar caused by the traditional open surgical approach affects the quality of life of patients [1]. At the end of the twentieth century, Gagner [2] and Huscher [3] performed the first endoscopic thyroidectomy, which pioneered the use of scarless neck surgery for treating thyroid tumors. Subsequently, various endoscopic thyroid procedures through the oral cavity, areola, axilla, subclavian, and retroauricular approaches were developed, and their combinations were investigated [4–9]. Depending on the method of establishing the cavity, this surgery can be divided into two types: traditional CO2 insufflation and gasless endoscopic thyroidectomy. Because of its unique advantages, noninflatable thyroid surgery is gradually being favored by surgeons. Currently, the gasless transaxillary procedure is widely preferred by physicians and patients. A recent multicenter survey in Korea reported that 66.7% of endoscopic thyroidectomy procedures are performed using the gasless transaxillary approach [10]. However, this procedure does not meet the clinical needs of patients who require total thyroidectomy, and in this situation, the transareolar procedure can be an effective option. An additional issue is that no single instrument is compatible with multiple endoscopic thyroidectomy procedures. In this study, we introduced a gasless endoscopic-assisted instrument developed by our team; this instrument is compatible with both gasless transaxillary and “internal support” gasless transareolar thyroid lumpectomies (the latter has rarely been reported). Here, we describe the application of our developed instrument in 180 patients who underwent endoscopic thyroidectomy procedures through transaxillary and transareolar approaches.

## Methods

### General information

#### Inclusion criteria

The following inclusion criteria were considered: (1) patients who were diagnosed or highly suspected to have unilateral papillary thyroid carcinoma (PTC) by fine needle aspiration biopsy; patients without a clear preoperative diagnosis, but whose intraoperative frozen section diagnosis was PTC; (2) patients who met the indications for unilateral lobectomy + isthmus; and (3) patients requiring dissection of lymph nodes in the affected central region. All patients provided their written informed consent prior to the surgery. The characteristics of the two procedures were explained by the surgeon before surgery, and the patients and their families were free to choose according to their needs. The following preoperative information was collected from the patients: gender, age, BMI, education level, and presence of Hashimoto’s thyroiditis. The criteria for Hashimoto’s thyroiditis were positive thyroid peroxidase antibodies and thyroglobulin antibodies along with diffuse changes in the thyroid parenchyma as suggested by an ultrasound examination. This retrospective study was approved by the ethical review committee of Bengbu Medical College.

#### Exclusion criteria

The following types of patients were excluded from the study: (1) patients requiring bilateral thyroidectomy; (2) patients requiring cervical lymph node dissection on the healthy side; (3) patients with a history of head and neck radiotherapy or thyroid surgery; (4) patients showing thyroid carcinoma invasion of adjacent organs such as the recurrent laryngeal nerve (RLN), esophagus, trachea, large blood vessels in the neck, or metastasis to clear lateral lymph nodes in the neck; (5) patients with a history of neck or chest surgery, scarring, or abnormal sensation; and (6) patients lost to follow-up.

### Surgical approach

Of 180 patients with unilateral PTC, 130 patients underwent gasless transaxillary endoscopic thyroidectomy (group A), while 50 patients underwent gasless transareolar endoscopic thyroidectomy (group B). The extent of surgical resection was defined as “thyroid lobe + isthmus + ipsilateral central lymph node dissection.” The surgery was performed using the same set of endoscopic-assisted instruments developed by our team (Patent Number: ZL201720223158.4; Anhui AOFO Medical Equipment Company, Anhui, China).

#### Structure and function of the developed instruments

**Fig. 1:**
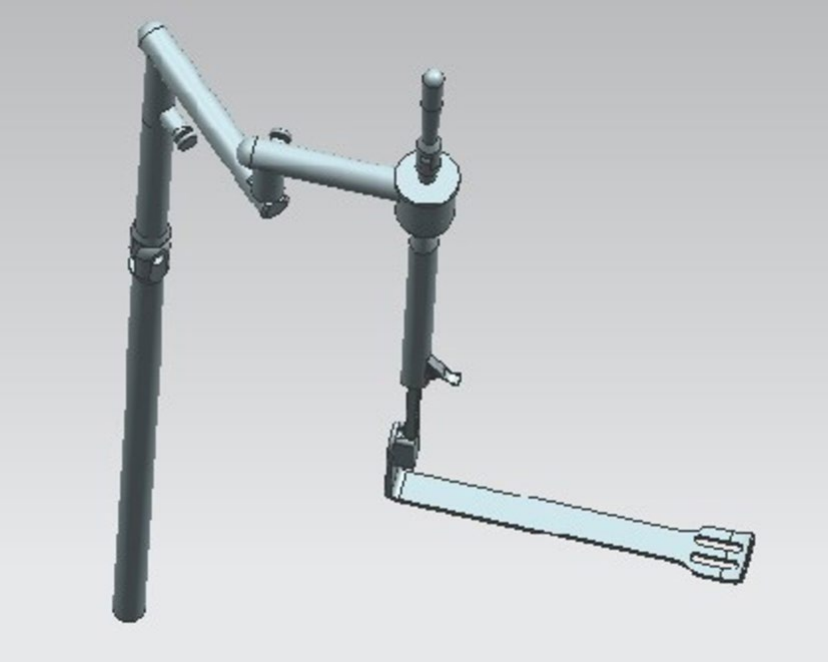
Schematic of the structure of the designed instruments

**Fig. 2:**
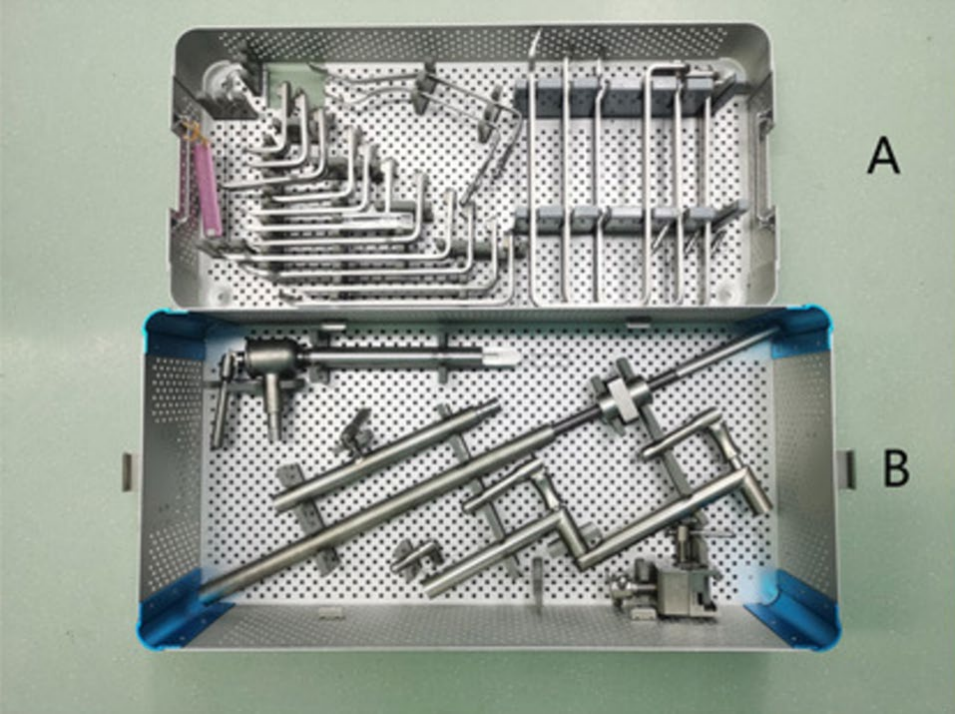
A set of gasless endoscopic-assisted instruments. **A** Four different types of retractors. **B** Main part of the instrument

**Fig. 3:**
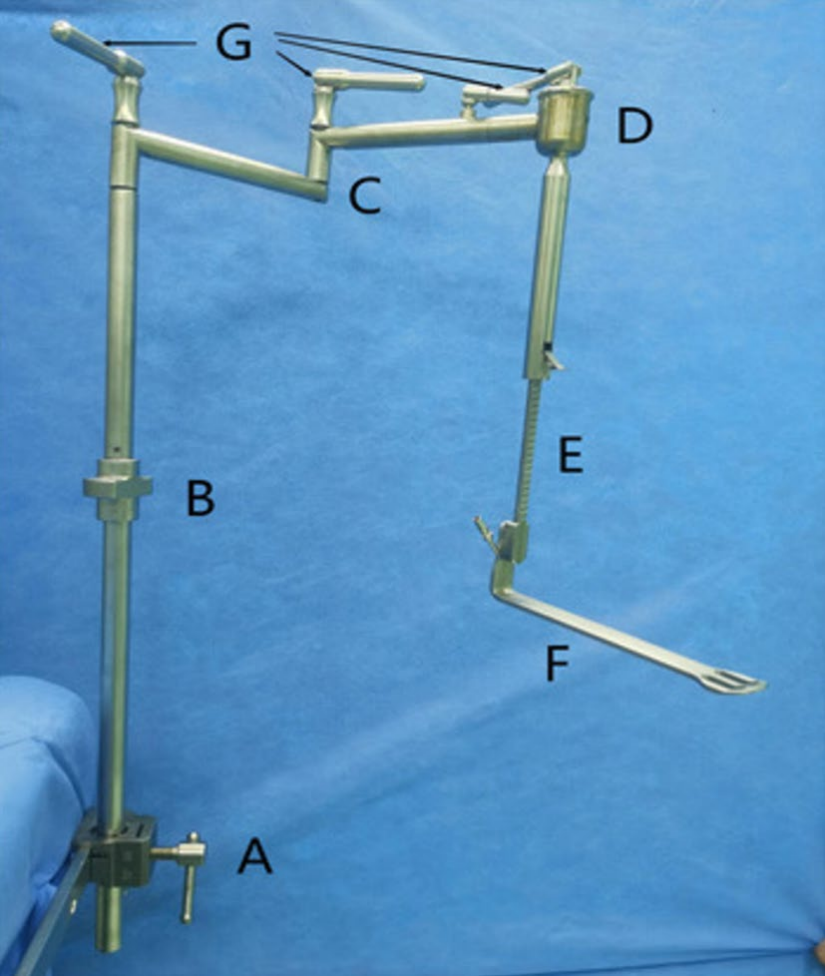
Physical diagram of the instruments. **A** Adjustable slider. **B** Height adjustment structure. **C** Horizontal structure. **D** Spherical structure. **E** Tissue tension trimmer. **F** Tissue retractor. **G** Joint locking structure

The set of instruments includes three components: a fixing device, an adjusting device, and a traction device (Fig. 1). ① The fixing device includes two parts: an adjustable slider and an upright rod. The slider fixes the upright rod on the operating table. The height of the upright rod can be adjusted through the telescoping mechanism. ② The adjusting device includes four structures with different dimensional attributes: vertical, horizontal, hemispherical (120°), and trimmer. The vertical device can adjust the height of the entire instrument during the operation. The horizontal device fits to patients with different body shapes by adjusting the width of the arm span of the instrument. The hemispherical device can adjust the angle of the goiter retractor. The trimmer device adjusts the tension of the tissue. Each adjustment device is equipped with a locking system for securing the entire device in a specific spatial position. ③ The traction device is designed with four different styles of goiter retractors to fit according to the needs of the different gasless endoscopic thyroidectomy procedures (Figs. 2 and 3).

#### Gasless transaxillary endoscopic-assisted thyroidectomy

The patient is placed in the supine position, with a cushion to elevate the shoulders, and the head is kept in the posterior supine position. The affected upper limb is horizontally abducted to 90° for anatomical markings. A 4–5 cm incision parallel to the skin line is made in the axillary crease. The surgical procedure is divided into two parts: establishment of the surgical space and thyroidectomy. The surgical operating space is created using a “three-step” approach. In the first step, the pectoralis major muscle is freed with an electric surgical knife assisted with an endoscopic light source after reaching the level of the sternocleidomastoid muscle, and a 5 mm trocar is placed in the anterior axillary line as an adjuvant surgical incision. In the second step, under the endoscopic light source, the space between the sternal and clavicular heads of the sternocleidomastoid muscle is found, and the omohyoid muscle is exposed. In the third step, the thyroid gland is exposed by separating the omohyoid muscle and the sternothyoid muscle, replacing the pull hook, and establishing a suitable surgical maneuvering space as required for the procedure. Thyroid surgery is generally performed with the lobe + isthmus combined with ipsilateral lymph node en bloc excision, which is similar to open surgery. First, the inferior parathyroid gland is searched, separated, and protected (if preservation is difficult, postoperative transplantation is performed). The RLN is revealed, separated, and protected to the extent possible in the direction of the entry to the larynx. Next, the lymphatic tissue in the pre-tracheal area is cleared, and the isthmus of the thyroid gland is dissected upward. The cricothyroid space is exposed by clearing the anterior laryngeal lymph nodes. The cricothyroid gap is found, and the supraglottic nerve is protected. The superior thyroid artery is separated with an ultrasonic scalpel to preserve the blood supply to the superior parathyroid gland.

**Fig. 4:**
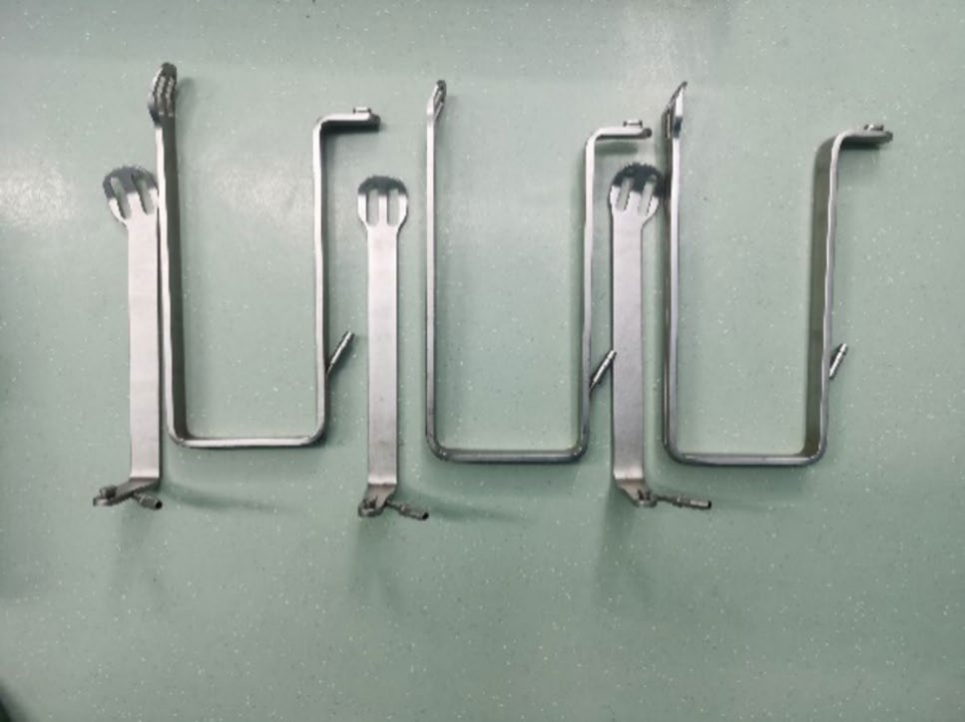
A special retractor for gasless transaxillary endoscopic thyroidectomy

**Fig. 5:**
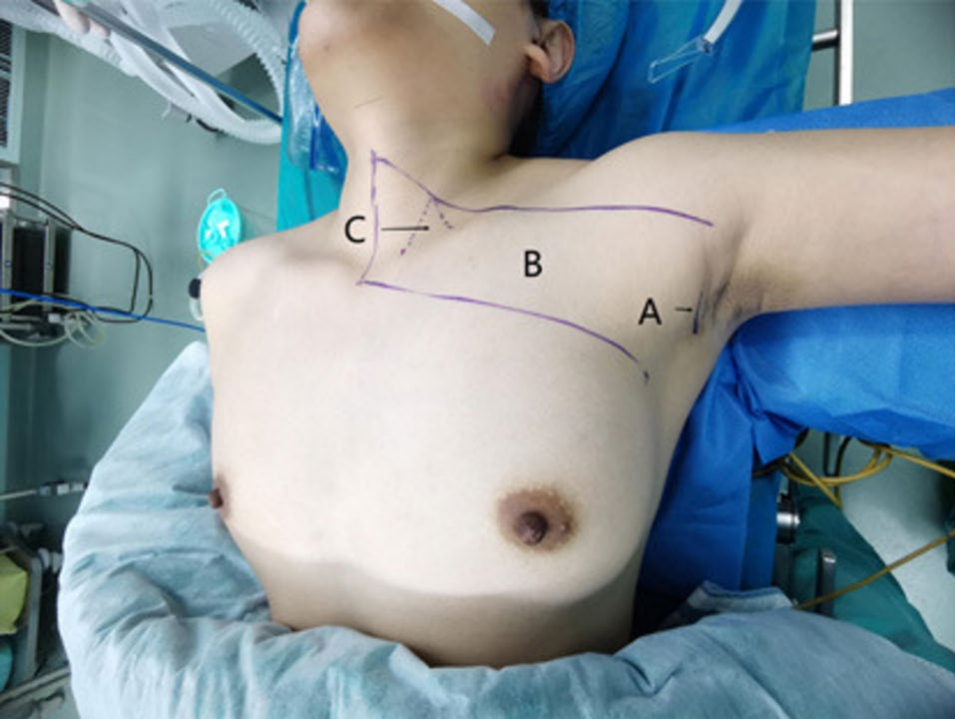
Surgical marks for gasless transaxillary endoscopic thyroidectomy. **A** Axillary incision. **B** Range of skin free of the cavity. **C** Position of the sternocleidomastoid intermuscular groove on the body surface

**Fig. 6:**
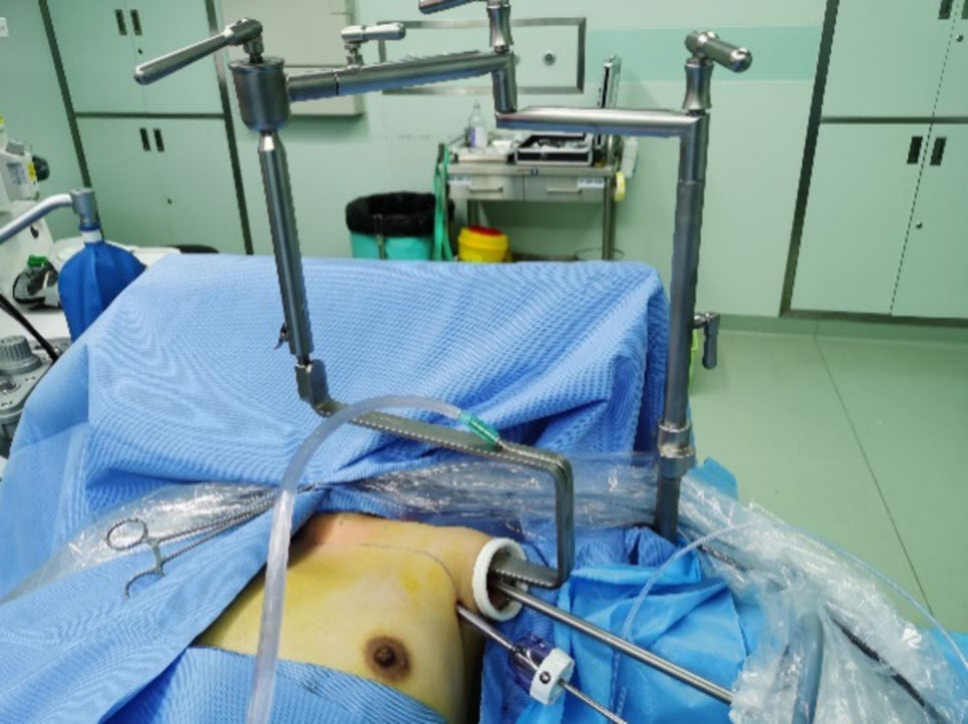
Intraoperative installation of the devices for gasless transaxillary endoscopic thyroidectomy

**Fig. 7:**
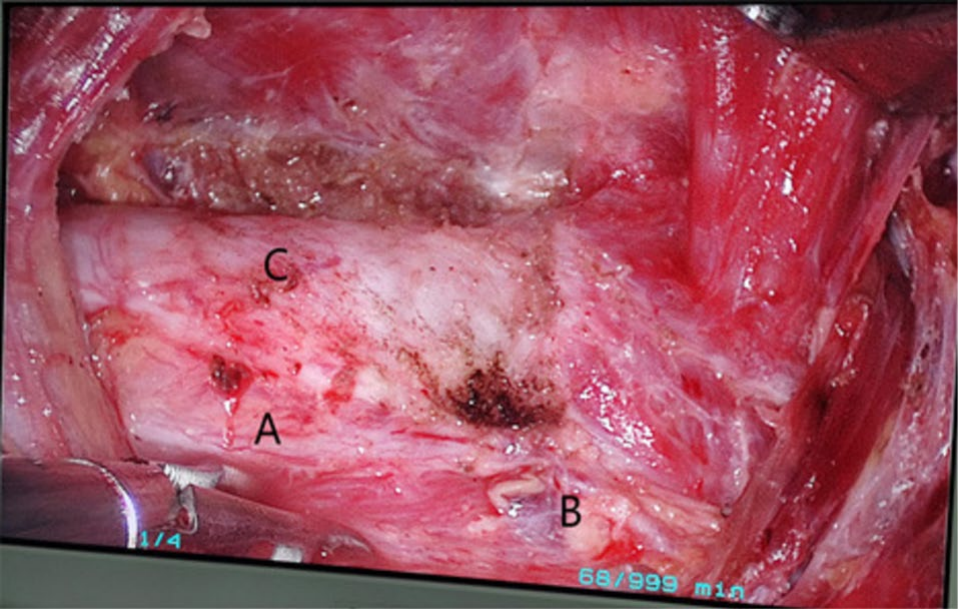
Effectiveness of radical treatment of thyroid cancer with gasless transaxillary endoscopic thyroidectomy. **A** Left recurrent laryngeal nerve. **B** Superior parathyroid gland. **C** Trachea

**Fig. 8:**
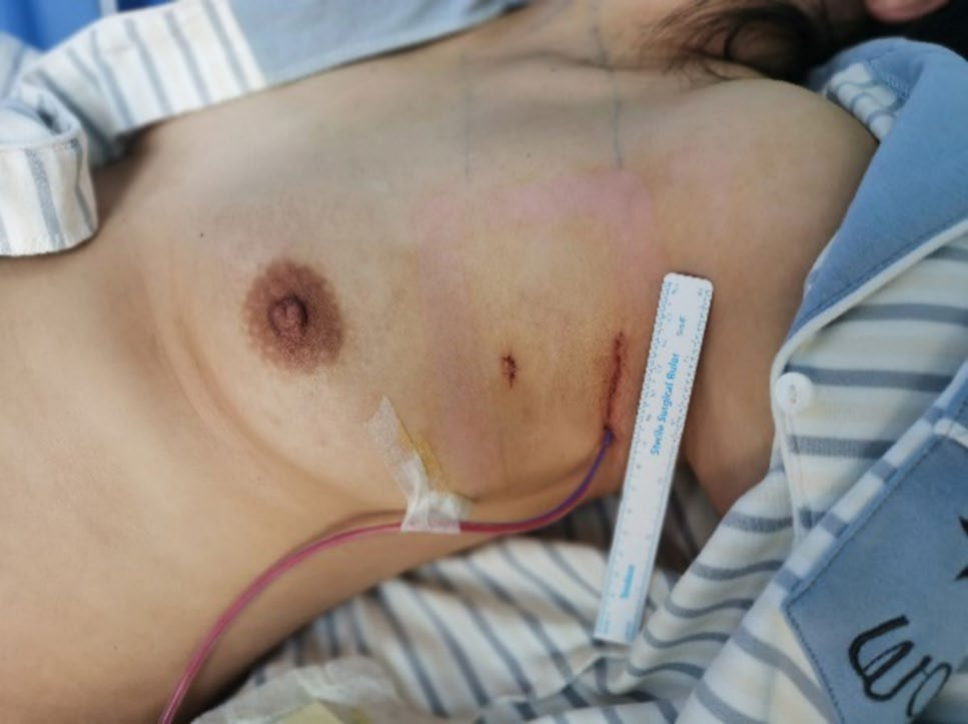
Postoperative results

Finally, the thyroid gland and its lymphatic drainage area were pushed together to the front of the trachea to reveal the laryngeal nerve. The berry ligament was dissociated with an electric hook. The lymphatic tissue specimen was sent to another laboratory for pathological examination (Figs. 4, 5, 6, 7, 8).

#### Gasless transareolar endoscopic-assisted thyroidectomy

**Fig. 9:**
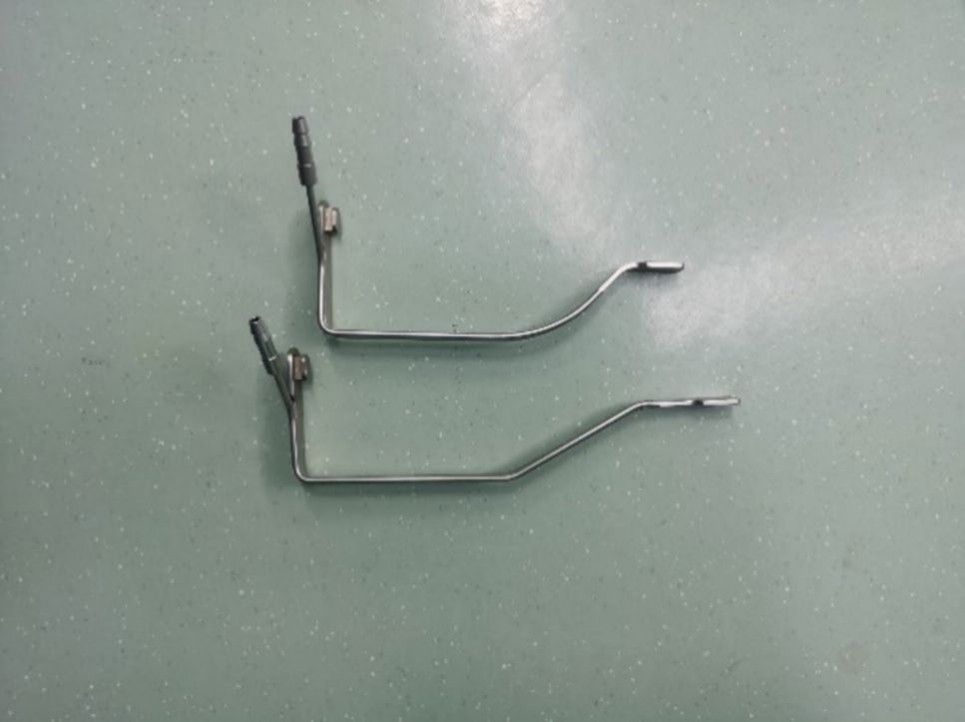
A special lumpectomy puller for gasless transareolar endoscopic thyroidectomy

**Fig. 10:**
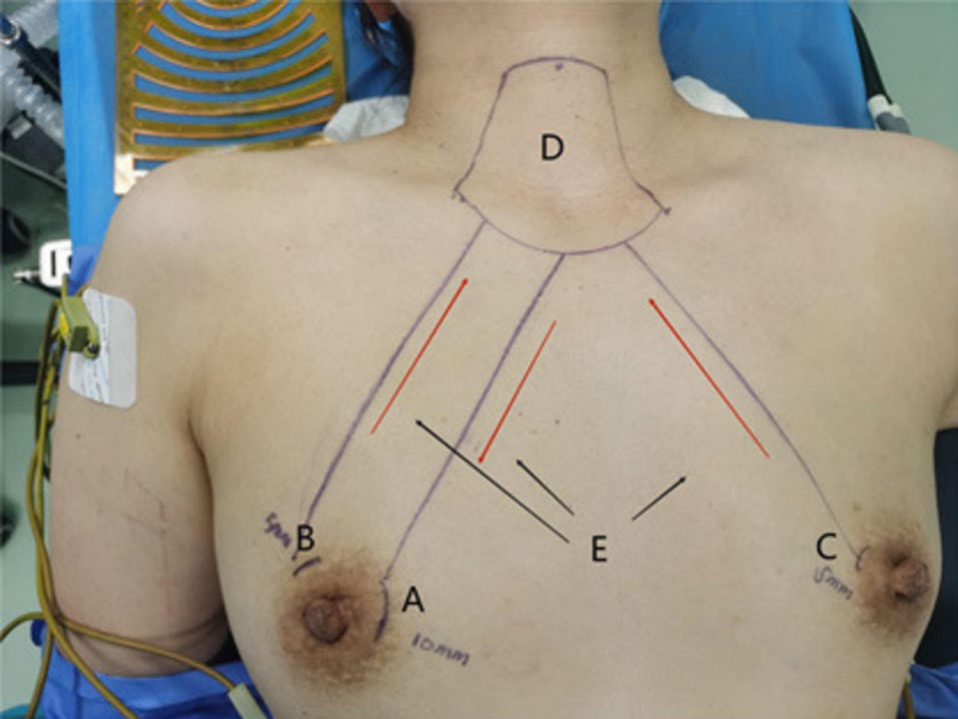
Surgical marks. **A** Observation port (discharge). **B** and **C** Operation port (intake). **D** Building a cavity with a skin-free range. **E** The direction of airflow in the operating cavity

**Fig. 11:**
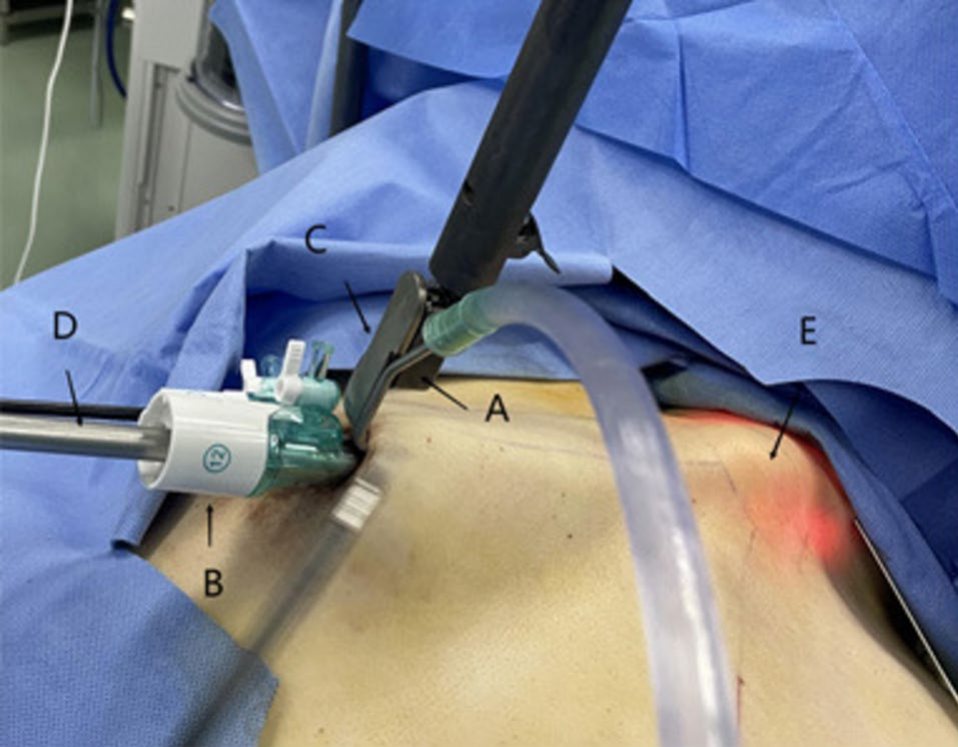
Intraoperative mounting operation for gasless transareolar endoscopic thyroidectomy. **A** Suction device on the retractor (vent). **B** A 12 mm trocar (observation hole) parallel to the retractor in the same subcutaneous tunnel. **C** Internally supported pre-tractor. **D** Endoscopic thyroidectomy lens. **E** A tissue retractor in the subcutaneous tunnel

**Fig. 12:**
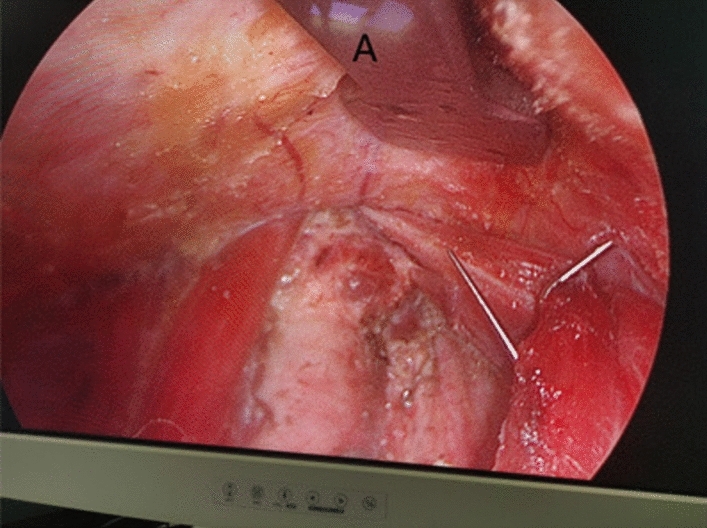
Effect of building a cavity with an internally supported pulling hook. **A** Internal retractor

**Fig. 13:**
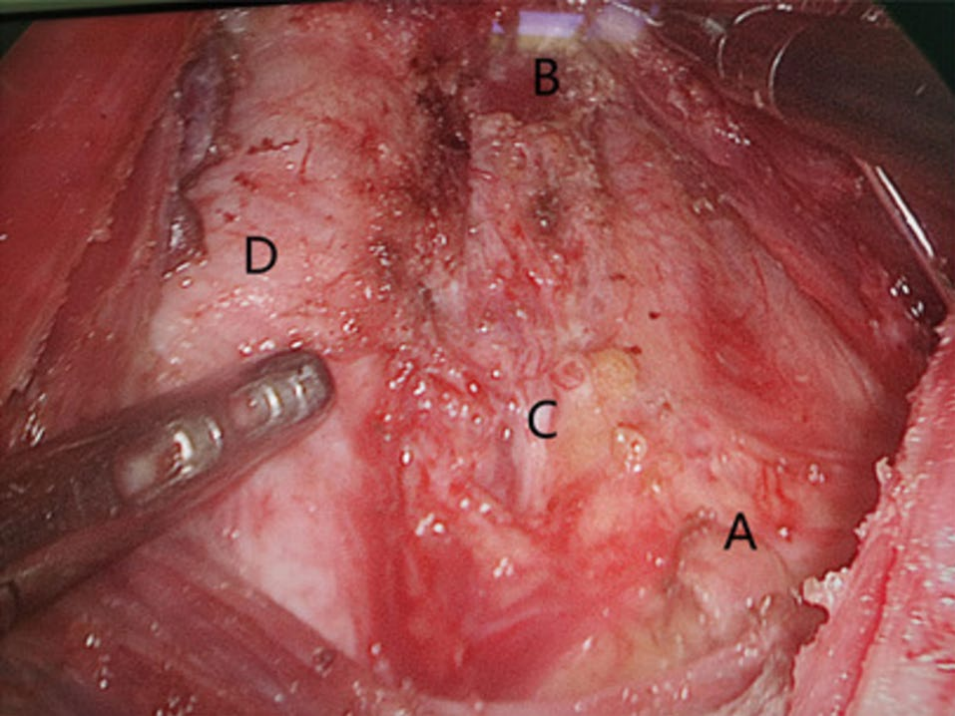
Results of radical treatment of PTC with gasless transareolar endoscopic thyroidectomy. **A** Inferior parathyroid gland. **B** Superior parathyroid gland. **C** Recurrent laryngeal nerve. **D** Trachea

**Fig. 14:**
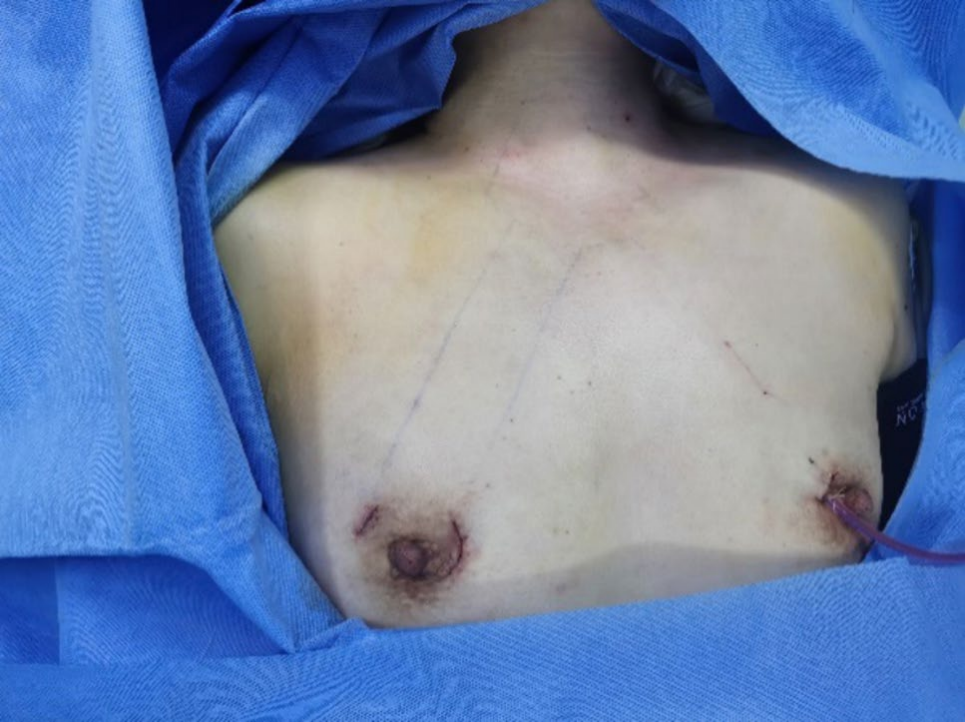
Postoperative effect

The patient is placed in the supine position, with a cushion to elevate the shoulders, and the head is kept in the posterior supine position. For anatomical markings by the surgeon, the affected upper limb is positioned vertically, while the lower limbs are kept as separated as possible.The surgical procedure is also divided into two parts: establishment of the surgical space and thyroidectomy. In the first part, the surgical space is established by applying a visual trocar to create a cross tunnel from the surface of the skin toward the sternocleidomastoid muscle, suspending 1–3 stitches of skin 4-0 silk at the crossover, freeing the subcutaneous tissue with an electric hook to a certain extent under endoscopy, placing a special gasless retractor, connecting a negative pressure suction device, and eliminating smoke interference. The freeing range is usually up to the level of the thyroid cartilage, with both sides up to the inner edge of the sternocleidomastoid muscle. In the second part, thyroid surgery is generally performed by the lobe + isthmus combined with ipsilateral lymph node dissection, with a slightly different procedure than that for the transaxillary approach. In the first step, the pre-tracheal lymphatic tissue is cleared, and the isthmus and the lymphatic tissue in the anterior laryngeal region are dissected. Subsequently, the cricothyroid space is entered. The superior laryngeal nerve is searched and protected. The superior thyroid vessels are dissected using ultrasonic knife branches. The inferior parathyroid gland is searched at the inferior level of the thyroid and protected (if preservation is difficult, a small amount of thyroid tissue can be preserved to reduce damage to the parathyroid blood supply). After repeatedly rinsing the wound with sterile distilled water, the cervical white line is closed with a 4-0 absorbable suture, and a drainage tube is placed to drain from the areola (Figs. 9, 10, 11, 12, 13, 14).

#### Postoperative treatment and follow-up

All patients received levothyroxine for TSH suppression at the postoperation stage. Thyroid function was measured at 1, 3, and 6 months postoperatively, and the drug dose was adjusted according to the risk of recurrence class based on the American Thyroid Association (ATA) guidelines. Patients with postoperative hoarseness underwent electronic laryngoscopy, and those whose symptoms did not completely resolve within 6 months were defined as having permanent RLN injury. A routine neck ultrasound examination was performed every 6 months postoperatively.

Postoperative quality of life was analyzed using both objective indicators and questionnaires containing subjective indicators. Patient satisfaction with neck appearance [Patient Satisfaction Scale (PSAS)] was assessed by outpatient review or telephone follow-up at 6 months postoperatively.

### Statistical analysis

To determine the clinical application value of the instruments developed by our team and the therapeutic advantages and disadvantages of the two surgical procedures for the same disease, 180 patients were assigned to two groups according to the surgical procedures they underwent. The clinicopathological characteristics, surgical outcomes, and complications were compared between the groups. Statistical analysis was performed using the chi-square test and t-test with SPSS 26.0 statistical software.

## Results

### Clinical characteristics

The clinicopathological characteristics of the 180 patients are summarized in Table 1. The mean age of the patients was 37.95 ± 9.70 and 37.50 ± 8.96 years in group A and group B, respectively (P = 0.90). The mean tumor size was 0.68 ± 0.46 and 0.71 ± 0.49 cm in group A and group B patients, respectively (P = 0.77). The BMI of the two groups was not significantly different (23.61 ± 4.07 vs. 24.24 ± 4.04, P = 0.30).

**Table 1:** Clinicopathological characteristics of the study patients

Characteristic	Group A (n = 130)	Group B (n = 50)	P
Gender			
Male	22 (16.9%)	12 (24%)	0.28
Female	108 (83.1%)	38 (64%)	
Age (years)	37.95 ± 9.70 95% CI (36.26–39.63)	37.50 ± 8.96 95% CI (34.95–40.05)	0.90
BMI (kg/m^2^)	23.61 ± 4.07 95% CI (22.90–24.31)	24.24 ± 4.04 95% CI (23.09–25.39)	0.30
Marital status	0.68 ± 0.46 95% CI (0.60–0.76)	0.71 ± 0.49 95% CI (0.57–0.85)	0.77
Unmarried			
Married	12 (9.2%)	5 (10%)	1.00
	118 (90.8%)	45 (90%)	
Education level			
High school and below	72 (55.4%)	35 (70%)	0.07
Specialized degree or above	58 (44.6%)	15 (30%)	
Hashimoto’s thyroiditis			
No	92 (70.8%)	35 (70%)	0.92
Yes	38 (29.2%)	15 (30%)	

Moreover, the two groups showed no significant differences in other clinical characteristics such as sex, marital status, educational status, and presence of Hashimoto’s thyroiditis (P > 0.05).

### Surgical outcomes and complications

Table 2 shows the surgical outcomes and complications in both groups. Because different paths were used for establishing the surgical cavity, the time of the establishment of the cavity also differed between the groups (35.62 ± 5.07 min in group A vs. 17.46 ± 2.55 min in group B, P < 0.05). The average time to establish the cavity was prolonged by approximately 15–20 min in group A. During cavity construction, venous bleeding occurred in 6 patients in group A. The surgical procedure in one of these patients was converted to open surgery because of difficulty in hemostasis; however, this difference between the two groups with regard to this aspect was not statistically significant (6/130 vs. 0/50, χ^2^ = 2.39, 0.19). Therefore, the risk of cavity formation in group A was slightly higher than that in group B. The two groups also showed no significant difference in the total operative time and postoperative hospital stay. The number of axillary lymphatic dissections was slightly less in group A than in group B; however, the difference was not significant (4.06 ± 0.26 vs. 4.52 ± 0.34, Z =  − 1.83, P = 0.07). Postoperative complications of vocal cord paralysis (4/130 vs. 2/50, P = 0.67) and dysphagia (24/130 vs. 5/50, P = 0.17) were similar in both groups. No patients with metastasis or recurrence were found in both groups during follow-up. With regard to postoperative satisfaction, both groups showed better cosmetic results on the PSAS, and no significant difference was observed between both groups (3.27 ± 1.52 vs. 3.28 ± 1.53, P = 0.97).

**Table 2:** Surgical outcomes and complications in group A and B patients

Characteristic	Group A (n = 130)	Group B (n = 50)	P
Establish cavity time (min)	35.62 ± 5.07 95% CI (34.74–36.50)	17.46 ± 2.55 95% CI (16.74–18.18)	0.00
Operate time (min)	145.54 ± 45.11 95% CI (137.71–153.37)	143.06 ± 46.70 95% CI (129.79–156.33)	0.65
Post-operative hospitalization time (days)	4.08 ± 1.48 95% CI (3.83–4.34)	3.72 ± 1.07 95% CI (3.42–4.02)	0.21
Number of lymph nodes dissected	4.06 ± 2.93 95% CI (3.55–4.57)	4.52 ± 2.38 95% CI (3.84–5.19)	0.07
Hemorrhage during cavi-building			
No	124 (95.4%)	50 (100%)	0.19
Yes	6 (4.6%)	0 (0)	
Vocal cord paralysis			
No	126 (96.9%)	42 (96%)	0.67
Yes	4 (3.1%)	2 (4%)	
Aglutition			
No	106 (81.5%)	45 (90%)	0.17
Yes	24 (18.5%)	5 (10%)	
Recurrence			
No	130 (100%)	50 (100%)	–
Yes	0 (0)	0 (0)	
Mark of PSAS	3.27 ± 1.52 95% CI (3.00–3.53)	3.28 ± 1.53 95% CI (2.85–3.71)	0.97

Group A = transaxillary group, Group B = transareolar group

## Discussion

Following the widespread use of endoscopic thyroidectomy in clinical practice, various approaches such as transoral, areolar, and axillary and their combinations have been developed. The advantages and disadvantages of each of these surgical approaches remain controversial. Endoscopic surgery is reported to achieve good results in terms of both surgical safety and cosmetic appearance [11–14]. Regardless of the approach used for endoscopic thyroidectomy, adequate surgical space needs to be established to meet the requirements of the surgery. The traditional cavity-building model relies on the use of CO2 at a certain pressure to maintain the surgical space; this approach has some limitations: intraoperative hypercapnia, obstruction of the return of blood from the brain to the heart, unstable maintenance of space, interference of smoke, and wastage of medical resources. In contrast, the gasless endoscopic thyroidectomy has many advantages: stability of space, clear visualization, flexible adjustment, less environmental pollution in the operating room, and resource saving [15]. However, each gasless procedure often requires a specific surgical instrument, which can easily lead to medical waste and hinder the widespread application of this technique. Consequently, there is an urgent need to develop cavity-building instruments that are compatible with multiple gasless procedures to meet clinical requirements.

In the present study, our team developed a set of instruments for constructing endoscopic-assisted cavity. The instrument design involved the combination of three different directional devices: vertical, horizontal, and hemispherical. By adjusting and fixing the device, it was possible to change the end of the hook to the specific spatial location required for surgery. We then clinically confirmed that this set of instruments was compatible with the gasless transaxillary and transareolar procedures. The surgical time, postoperative hospital stay, number of lymph node excised, and complications were similar to those reported in the literature [8, 11]. Lateral cervical lymph node dissection and transoral/sub-chin thyroid surgery are also being used in clinical practice. Based on the results of clinical practice, this set of instruments can meet the requirements of various approaches used for gasless endoscopic thyroidectomy. These instruments are simple to operate, flexible to adjust, stable in space, and provide ample support.

Since the development of the anterior mammary approach for endoscopic thyroidectomy by Ohgami et al. [16], this procedure has become the most classical approach with advantages in managing bilateral total thyroid dissection. According to the available literature, there is no significant difference in the number of lymph nodes cleared, positive lymph nodes, and lymph node metastases between thyroidectomy with the transmammary approach and conventional open surgery. This finding suggests that central lymph node clearance through the anterior mammary approach is safe [6, 17]. A potential issue with endoscopic neck surgery is the complication of CO2 inhalation. Gagner [2] and Gottlieb et al. [18] reported severe subcutaneous emphysema and hypercapnia in the early stages of parathyroid endoscopic surgery through CO2 insufflation. The present study is the first to report the use of a gasless, inward-supported transthoracic breast approach for thyroid endoscopy. A tent-like space is created subcutaneously by placing a retractor through the tunnel of the trocar in the areola, and a suction device on the retractor is used to allow air circulation in the “tent” to lower the intracavitary temperature and remove the smoke. The application of this technique in clinical practice confirmed that it has clear images, less smoke interference, stable cavity construction, flexible operation, no CO2-related complications, and reduced wastage of medical resources. The end of the hook is a flat design with a wide contact area with the skin, and the skin pressure is low. Postoperative observation showed no complications such as localized flap ischemia and necrosis.

The main advantages of the axillary approach for thyroid surgery are good cosmetic outcomes and high patient acceptance. Ikeda et al. [19] (inflated) and Kang SW et al. [8] (noninflated) presented preliminary results of the axillary approach for thyroid surgery in 2001. These findings demonstrate that the procedure can be performed successfully, with easier intraoperative identification of the ipsilateral RLN, elimination of the central lymph nodes, preservation of the parathyroid glands, and minimal complications. This surgical pathway technique has been well validated in subsequent studies [19–23]. However, several factors limit the general use of the axillary approach, such as difficulties in the dissection and resection of the contralateral thyroid lobe, insufficient working space, and the need for special equipment. The axillary approach provides a lateral anatomical view of the thyroid gland, which is unfamiliar to most surgeons and requires skilled surgical training [11, 24]. In the present study, a significant difference in cavity building time was observed between both groups, with significantly longer cavity building time in the transaxillary group than in the transareolar group. This may be related to the following factors: the long distance to build the cavity tunnel, the time required to find the intramuscular sulcus of the sternocleidomastoid muscle, the separation of the strap muscles, the need to change the position of the pulling hook intraoperatively, etc. The risk of cavity construction is also higher in the transaxillary group than in the transareolar group, with six cases of venous bleeding in the present study, wherein one of them was converted directly into an open surgical procedure. In contrast, the anatomical level of the transareolar cavity is simple, clear, and easily identifiable and involves less critical tissue structures; consequently, it is faster and less risky. Based on the advantages of the existing instruments, our team repeatedly improved and conducted clinical trials of the cavity building instruments to develop different models of axillary cavity building hooks. These models can complete the surgical operation through a hidden incision of approximately 3 cm in the axillary fold, with convenient intraoperative adjustment, flexible replacement, and spatial stability.

In the present study, we observed RLN palsy in 4 (3.1%) and 2 (4%) patients in the transaxillary and transareolar groups, respectively; this finding is similar to that reported in previous studies [25, 26]. RLN damage is a common complication in thyroid surgery, and it may be caused by the following factors: use of a high energy instrument, proficiency of the surgeon, and presence of Hashimoto’s thyroiditis. The application of ultrasonic knives in the endoscopic group, particularly for removing the thyroid gland at the entrance of the larynx, increases the risk of thermal conduction injury. The use of endoscopic bipolar electrocoagulation has reduced the incidence of such injuries. Therefore, maintaining a safe distance for high energy instruments and improving the overall safety of surgical instruments are the key factors to reduce RLN-associated injury in endoscopic surgery. The proficiency of the surgeon is another factor leading to RLN damage. Hurtadolopez et al. [27] concluded that the detection and exposure of the nerve during thyroid surgery is an effective approach to avoid injury. RLN damage could also be caused by the concomitant presence of Hashimoto’s thyroiditis. In such patients, the thyroid gland is more fragile and hard and has severe adhesions, which cause bleeding easily during the operation. Thus, it is particularly difficult to reveal and protect the RLN, which can be easily damaged [28, 29].

In the present study, the total number of lymph node clearance in group A was slightly lower than that in group B (4.06 ± 0.26 vs. 4.52 ± 0.34, P = 0.07); however, no recurrent cases were found during follow-up. Analysis of the intraoperative field exposure showed that both groups had similar extent and completeness of clearance. This result may be related to the clearance of the lymph nodes in the posterior region of the right RLN and the anterior region of the trachea. Both these regions have some operational difficulties and visual blindness in the transaxillary approach as compared to that in the transareolar approach. However, for low-risk PTC, the extent of clearance does not affect the prognosis of patients.

Some researchers [30] have reported that patients’ choice of scarless neck surgery depends on the perception of the scar, their image requirements, psychological needs, and recommendations of physicians. Our present study revealed that in addition to highly educated and unmarried patients, an increasing number of patients with a low level of education and married patients have gradually begun to accept endoscopic surgery; this finding agrees with a previous report [31]. This shows that patients have specific requirements for postoperative quality of life, and hence, further development of thyroid endoscopy is becoming increasingly important.

The present study has some limitations such as insufficient sample size and short follow-up time.

## Conclusion

Gasless endoscopic thyroidectomy is a promising technique, and the set of endoscopic-assisted instruments that we developed is suitable for meeting the requirements of gasless endoscopic surgery through the axilla and areola. Both procedures have a high level of safety, and each has advantages and disadvantages; thus, the optimal approach should be selected according to the different needs of individual patients.

## Data Availability

All original data in this paper were provided by the authors and have not been improperly retained.
